# Cross-Reactive Effects of Vaccines: Heterologous Immunity between Tetanus and Chlamydia

**DOI:** 10.3390/vaccines8040719

**Published:** 2020-12-01

**Authors:** Marijana Stojanovic, Ivana Lukic, Emilija Marinkovic, Ana Kovacevic, Radmila Miljkovic, Joshua Tobias, Irma Schabussova, Mario Zlatović, Talin Barisani-Asenbauer, Ursula Wiedermann, Aleksandra Inic-Kanada

**Affiliations:** 1 Institute of Virology, Vaccines, and Sera–TORLAK, 11152 Belgrade, Serbia; mstojanovic@torlak.rs (M.S.); ilukic@torlak.rs (I.L.); emilija.marinkovic@mailbox.tu-dresden.de (E.M.); afilipovic@torlak.rs (A.K.); rmiljkovic@torlak.rs (R.M.); 2 Center for Pathophysiology Infectiology and Immunology, Institute of Specific Prophylaxis and Tropical Medicine, Medical University of Vienna, 1090 Vienna, Austria; joshua.tobias@meduniwien.ac.at (J.T.); irma.schabussova@meduniwien.ac.at (I.S.); talin.barisani@meduniwien.ac.at (T.B.-A.); ursula.wiedermann@meduniwien.ac.at (U.W.); 3 Faculty of Chemistry, University of Belgrade, 11000 Belgrade, Serbia; mario@chem.bg.ac.rs

**Keywords:** vaccination, heterologous immunity, antibodies, tetanus, Chlamydia, cross-reactivity

## Abstract

Vaccines can have heterologous effects on the immune system, i.e., effects other than triggering an immune response against the disease targeted by the vaccine. We investigated whether monoclonal antibodies (mAbs) specific for tetanus could cross-react with Chlamydia and confer heterologous protection against chlamydial infection. The capability of two tetanus-specific mAbs, namely mAb26 and mAb51, to prevent chlamydial infection has been assessed: (i) in vitro, by performing a neutralization assay using human conjunctival epithelial (HCjE) cells infected with *Chlamydia trachomatis* serovar B, and (ii) in vivo, by using a guinea pig model of *Chlamydia* *caviae*-induced inclusion conjunctivitis. The mAb26 has been superior in comparison with mAb51 in the prevention of chlamydial infection in HCjE cells. The mAb26 has conferred ≈40% inhibition of the infection, compared to less than 5% inhibition in the presence of the mAb51. In vivo, mAb26 significantly diminished ocular pathology intensity in guinea pigs infected with *C. caviae* compared to either the mAb51-treated or sham-treated guinea pigs. Our data provide insights that tetanus immunization generates antibodies which induce heterologous chlamydial immunity and promote protection beyond the intended target pathogen.

## 1. Introduction

In the first edition of the textbook Vaccines (1988), Stanley Plotkin stated: “The impact of vaccination on the health of the world’s peoples is hard to exaggerate. With the exception of safe water, no other modality has had such a major effect on mortality reduction and population growth” [1]. Vaccines indeed have saved and are still saving millions of lives on Earth. Vaccination is the finest way of initiating protection against viral and bacterial infections; nevertheless, currently, only 26 vaccines against infectious diseases are licensed. Vaccines against many pathogens such as human immunodeficiency virus, Zika virus, hepatitis C virus, SARS-CoV-2, *Chlamydia trachomatis*, and *Borrelia burgdorferi* are still unavailable for a variety of reasons, which include but are not limited to (i) pathogen complexity, (ii) pathogen novelty, (iii) absence of efficient delivery system and adjuvants, (iv) lack of knowledge on the mechanisms of protective immune response induction, (v) lack of relevant/suitable animal models, and (vi) ethical issues for conducting clinical trials [2,3,4,5,6,7].

Data on heterologous effects of vaccination on adaptive immunity, either beneficial or detrimental, accumulate [8,9,10,11,12,13]. It has been shown that vaccination established a protective immune response against a specific microbe could also result in heterologous immunity, i.e., an immune response directed against another unrelated (heterologous) pathogen [12,14]. The mechanisms underlying the establishment of heterologous immunity are far from being fully understood but are thought to be based, at least in part, on the cross-protection of cross-reactive antibodies [15]. Cross-reactivity of an antibody implies its ability to interact with a heterologous antigen, which possesses a certain degree of structural similarity to the primary antigen, in addition to the antigen against which it was initially generated (a primary antigen).

Bacteria of the genus Chlamydia are obligate intracellular bacteria characterized by a biphasic life cycle. In the form of the infectious and metabolically inactive elementary body (EB), Chlamydia infects the host cell. Once inside, the EB transitions into a non-infectious, metabolically active, and replicating reticular body (RB). Following multiplication, RBs are back-transformed into EBs, and after lysis of the host cell, EBs are ready to start a new infectious cycle [16,17]. It has been shown that neutralizing antibodies specific for proteins expressed on the surface of EBs [5,18] have a significant role in preventing the initial contact of EBs with host cells [19,20,21]. Chlamydia-specific antibodies, primarily locally at the infection site, are known to be important for controlling and thus preventing the spread of chlamydial infection [5,22,23,24,25,26,27,28,29].

*Clostridium tetani* is an anaerobic bacterium and is the causative agent of tetanus disease. *C. tetani* produces a neurotoxin, tetanus toxin (TeNT) [30], which induces death at very low concentrations (2.5 ng/kg) [31]. The protection against tetanus disease is acquired after vaccination with TeNT derivative, tetanus toxoid (TTd) [32,33]. Interestingly, the vaccination of healthy volunteers with tetanus vaccine and other non-chlamydial antigens led to the production of antibodies recognizing chlamydial antigens, suggesting structural homology between tetanus and certain chlamydial antigens [34].

We have previously produced and characterized eight anti-tetanus monoclonal antibodies (mAbs) [35,36,37,38] and further established an in vitro selection system, which predicts the success of protection induced by these antibodies in vivo [35].

In the present study, we address the question of the heterologous recognition of chlamydial antigens by tetanus-specific antibodies. Here, we report data that certain tetanus-specific antibodies confer partial heterologous protection against chlamydial infection in vitro and most importantly in vivo.

## 2. Materials and Methods

### 2.1. Protein Sequence Alignments

For comparisons between the protein sequences of TeNT and the chlamydial proteins, the Basic Local Alignment Search Tool (BLAST) (Altschul et al., 1990 & 1997) was used. We used the default scoring matrix for the BLASTp-BLOSUM62 (BLOcks SUBstitution Matrix 62) matrix. A Blocks Substitution Matrix is a substitution scoring matrix in which scores for each position are derived from observations of the frequencies of substitutions in blocks of local alignments in related proteins. In the BLOSUM62 matrix, the alignment from which scores were derived was created using sequences sharing no more than 62% identity. Sequences more identical than 62% are represented by a single sequence in the alignment to avoid over-weighting closely related family members. In our study, a protein query sequence (the input sequence to which other sequence was compared) was the protein sequence of TeNT and subject protein sequences of chlamydial proteins, which are available on the sequence database UniProt (https://www.uniprot.org).

A BLAST search starts with finding a perfect sequence match of length given by word size. Then, this initial region of an exact sequence match is extended in both directions, allowing gaps and substitutions based on the scoring thresholds. Using the BLOSUM62 matrix, we calculated the Score (S) value that represents the highest alignment score between the query sequence and the database sequence segment. A positive score is given to the more likely substitutions (amino acids with similar chemical properties) while less likely substitutions or gaps in a sequence diminish the S value. The Expectation value or Expect value (E value) represents the number of different alignments, which is expected to occur in a database search by chance: the lower the E value, the more significant the score and the alignment.

### 2.2. Antigens

Stocks of *Chlamydia trachomatis* strain HAR-36 (ATCC^®^ VR-573™, Manassas, VA, USA; *C. trachomatis* serovar B) and *Chlamydia caviae* (a natural chlamydial pathogen in guinea pigs) kindly provided by Prof. Roger G. Rank were prepared according to standard methodology in McCoy cells [39] and frozen at −80 °C in the sucrose–phosphate–glutamate buffer (SPG) until needed.

### 2.3. Tetanus-Specific Monoclonal Antibodies

Eight murine anti-tetanus monoclonal antibodies (mAbs) produced by us and previously well-characterized [35,36,37,38] were preliminarily tested for binding to *C. trachomatis* serovar B and *C. caviae*. In brief, mAb26 binds TeNT and TTd with low affinity and does not confer protection against tetanus disease. At the same time, mAb51 possesses a high affinity for TeNT and TTd, and, when passively infused, fully protects mice from TeNT intoxication [36]. After validation that mAb26 and mAb51 bind *C. trachomatis* serovar B and *C. caviae* in a direct ELISA, these two mAbs were selected for further research.

### 2.4. Inhibition of mAbs Binding to Tetanus by Chlamydia

Evaluation of mAbs’ potential to cross-react with chlamydial antigens was performed using a competition/inhibition ELISA. Microtiter plates (MaxiSorp; Nunc, Roskilde, Denmark) were coated (50 μL/well) with TTd (1 μg/mL TTd in phosphate-buffered saline (PBS)) by overnight adsorption at 4 °C. The plates were blocked with 1% bovine serum albumin (BSA) in PBS (*w*/*v*) for 2 h hours at RT. The blocking, as well as all subsequent ELISA steps were followed by washing (4 × 200 μL/well) with 0.05% Tween 20/PBS (*v*/*v*). Then, the samples (preincubated for 1 h at room temperature (RT)) containing *C. trachomatis* serovar B and *C. caviae* in increasing concentrations (0–1 × 10^7^ infectious forming units (IFU)/mL in a 10-fold dilution) and premixed individually with mAb26 and mAb51 (1 μg/mL, final concentration) prepared in 1% BSA/PBS (*w*/*v*), were added to the plates (50 μL/well) and incubated for 1 h at RT. The mAb’s binding was detected with biotin-labeled anti-mouse immunoglobulin G (IgG) (Sigma-Aldrich, Steinheim, Germany). The ExtrAvidine-peroxidase/o-phenylenediamine system (Sigma-Aldrich, Steinheim, Germany) was used for visualization. The absorbance was read at 492/620 nm. Solutions containing only the mAbs in increasing concentrations (up to 1 μg/mL) were treated in the same manner and were used as standards. The percentage of inhibition for each sample was calculated from the standard curve where the absorbance (A492/620) was plotted versus mAbs concentrations.

### 2.5. In Vitro Neutralization Assay—A Percentage of Neutralization

A human conjunctival epithelial (HCjE) cell line was maintained in keratinocyte serum-free medium (KSF medium; Life Technologies, Paisley, UK) at 37 °C with 5% CO_2_ and 95% humidity. The cells were passaged at 70% confluency and the trypsinization (0.05% Trypsin/0.02% ethylenediaminetetraacetic acid in PBS-PAA Laboratories GmbH, Pasching, Austria) was used for cells harvesting. Then, cells were seeded at a density of 70,000 cells/well in 8-chamber slides (Millipore, Billerica, MA, USA) and allowed to adhere overnight. Serial dilutions of *C. trachomatis* serovar B ranging from 1 × 10^3^ to 1 × 10^7^ IFU/well were incubated with the individual mAbs (5 μg/mL) in SPG for 2 h at 37 °C. Respective serial dilutions of *C. trachomatis* serovar B in SPG without mAbs served as controls. After incubation, all samples were centrifuged at 14,000× *g* for 10 min, and the resulting pellets were resuspended in inoculation medium (1:1 Dulbecco’s Modified Eagle Medium/Ham’s F12 supplemented with 25 mM HEPES; Life Technologies, Paisley, UK) and added to HCjE cell cultures. The slides were spun for 1 h at 2000× *g* and then incubated for an additional hour at 37 °C. Following incubation, the inoculation medium was changed to the chlamydial growth medium for HCjE cells (KSF medium supplemented with 5 mg/mL gentamycin and 25 mg/mL vancomycin; Life Technologies, Paisley, UK) and left for incubation at 37 °C for 48 h.

Cells were fixed with ice-cold methanol and stained with fluorescein isothiocyanate (FITC)-conjugated mAb against Chlamydia lipopolysaccharides (LPS) (Clone B410F, Pierce Biotechnology, Rockford, IL, USA). As a counterstain, we used the 4′, 6-diamidino-2-phenylindole (1 μg/mL; Sigma Aldrich, St. Louis, MO, USA). A fluorescence microscope (Axio-Observer, Zeiss, Vienna, Austria) was used for counting the number of inclusions. Image acquisition was carried out using TissueFAXS software, v.4 (TissueGnostics, Vienna, Austria).

### 2.6. Ethics Statement

The Ethics Committee for the Welfare of Experimental Animals at the Institute of Virology, Vaccines, and Sera-Torlak approved animal experiments used in this study (Approval Number 011-00-00510/2011-05/5). Experiments conformed to the Basel Declaration that is committed to the 3Rs (Replacement, Reduction and Refinement) principle. The guinea pigs were observed daily by a veterinarian. Euthanasia was carried out by lethal CO_2_ overdose.

### 2.7. Experimental Animals

Six-week-old Hartley strain female guinea pigs weighing 300 ± 35 g (five animals per group) were housed individually in cages at the Animal Facility of the Institute of Virology, Vaccines, and Sera-TORLAK and kept at a temperature of 21 °C with access to water and food ad libitum. The conjunctiva-associated lymphoid tissue (CALT) of guinea pigs, which is very similar to the organization of CALT in humans, brands the guinea pig inclusion conjunctivitis model the most relevant animal model after non-human primates to investigate chlamydial infection in vivo.

### 2.8. C. caviae Conjunctival Infections

On day 0, 25 μL of SPG buffer containing (i) 1 × 10^4^ IFU of *C. caviae*–positive control, (ii) 1 × 10^4^ IFU of *C. caviae* preincubated with 10 μg/mL mAb26, and (iii) 1 × 10^4^ IFU of *C. caviae* preincubated with 10 μg/mL mAb51 were applied directly into the conjunctival sac of anesthetized guinea pigs. The dose was chosen according to previously established criteria [40,41,42]. The control group of animals (sham control) received SPG buffer only. During the acute phase of infection (first 7 days) [41], guinea pigs were treated with mAb26 and mAb51 (25 μL of 10 μg/mL mAb in PBS). None of the applied formulations caused changes in behavioral patterns in the treated animals compared to their respective controls nor disturbed their usual daily activity. Guinea pigs’ eyes were monitored daily by visual scoring of gross ocular pathology during the post-infection period.

### 2.9. Pathology Scoring

The guinea pigs’ eyes were daily examined by visual scoring of gross ocular pathology and graded as described previously [41,43].

### 2.10. Statistical Analysis

The observed differences were evaluated for statistical significance using a one-way ANOVA test followed by Bonferroni post-test. A probability (*p*) value of 0.05 was set as the significance threshold. All statistical analyses were performed with the GraphPad Prism 6.0 software (GraphPad Inc., La Jolla, CA, USA).

## 3. Results

### 3.1. Identification of Potentially Cross-Reactive Sequences between Tetanus and Chlamydial Proteins

We performed BLAST searches [44] and examined whether there is a structural similarity between TeNT and chlamydial proteins and to what extent. Chlamydial proteins, which were described in the literature as IgG-immunoreactive antigens, were selected (Table 1) [5,45,46,47,48,49,50].

The comparison was made at the level of short peptide sequences (up to 100 amino acids). Table 1 is showing the S and E values for chlamydial amino acid sequences with the best overlap with the amino acid sequence of TeNT as well as the length of the peptide sequence and the TeNT region with the maximum overlap.

**Figure 1:**
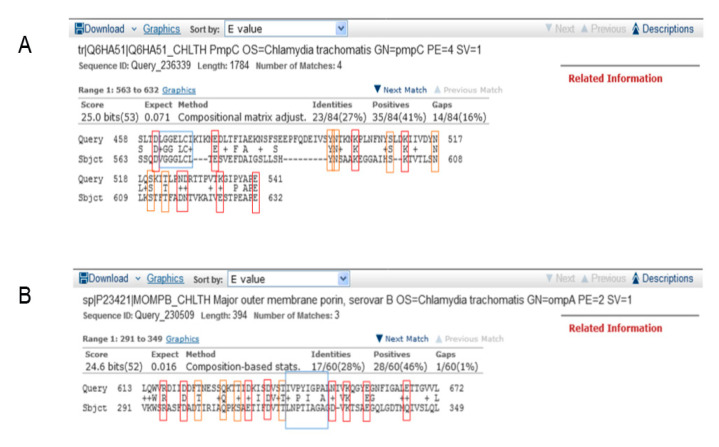
Amino acid sequence homology of the short peptide sequences of chlamydial proteins PmpC and MOMP-B with the tetanus toxin (TeNT). The parameters on the BLASTp network server were automatically set. The maximum overlaps of the TeNT amino acid sequence (Sbjct) and amino acid sequences of (**A**) PmpC and (**B**) MOMP-B (Query) are shown. The polar-charged amino acids are marked in red, the polar-uncharged amino acids are marked in orange, and the hydrophobic amino acids are marked in blue.

The best overlap of the TeNT and chlamydial amino acid sequences was observed for the *C. trachomatis* polymorphic membrane protein C (PmpC) and the major outer membrane protein (MOMP) from *C. trachomatis* serovar B (MOMP-B). By comparing the amino acid sequences of PmpC vs. TeNT, we detected that there were four overlaps at the level of short peptide sequences and that the best overlap of the PmpC sequence is represented by 84 amino acids in length with the sequence within the heavy chain of TeNT. The E value for the given overlap is 0.071 (Figure 1A). The comparison of the amino acid sequences of MOMP-B vs. TeNT yielded an E value of 0.016 (Figure 1B) for the peptide sequence MOMP-B of length 60 amino acid residues with the peptide sequence of the TeNT heavy chain.

### 3.2. Cross-Reactivity of Anti-TTd mAbs and Chlamydial Proteins

**Figure 2:**
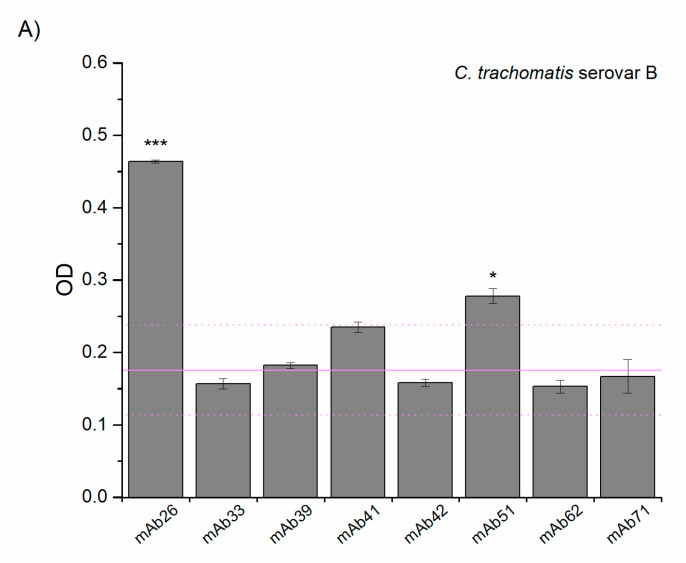

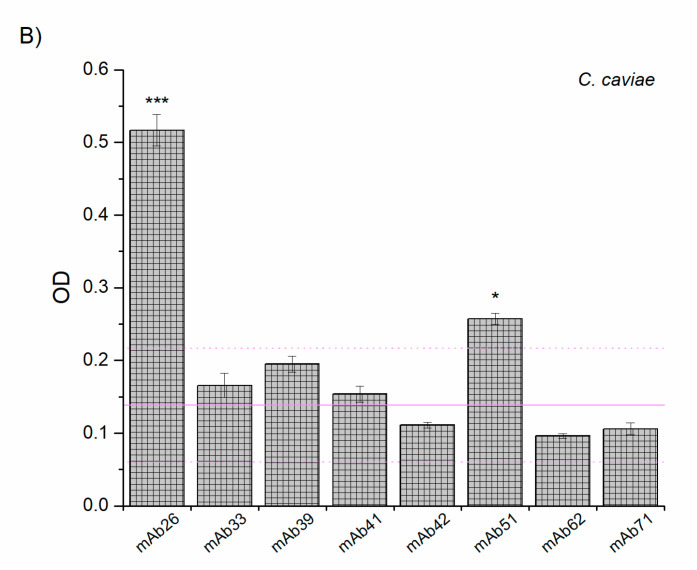
Reactivity of tetanus-specific monoclonal antibodies (mAbs) with *C. trachomatis* serovar B (**A**) and *C. caviae* (**B**). Threshold binding was determined with respect to mAbs other than mAb26 and mAb51 and presented as a mean +2SD (light magenta solid/doted lines). All samples were assessed in triplicate and results presented as a mean ± S.E; * *p* < 0.05, *** *p* < 0.001 compared to the threshold binding.

The cross-reactivity study of eight anti-TTd mAbs with *C. trachomatis* serovar B and *C. caviae* was performed by ELISA. As depicted in Figure 2, mAb26 showed the highest reactivity against both chlamydial species. The mAb51 showed lower reactivity in comparison to mAb26, but this reactivity was higher when compared to other tested anti-TTd mAbs. This finding, together with already known differences in the binding of mAb26 and mAb51 to TeNT (Table S1), was the reason behind selecting these two mAbs for further exploring the role of cross-reactivity in protection against chlamydial infection.

**Figure 3:**
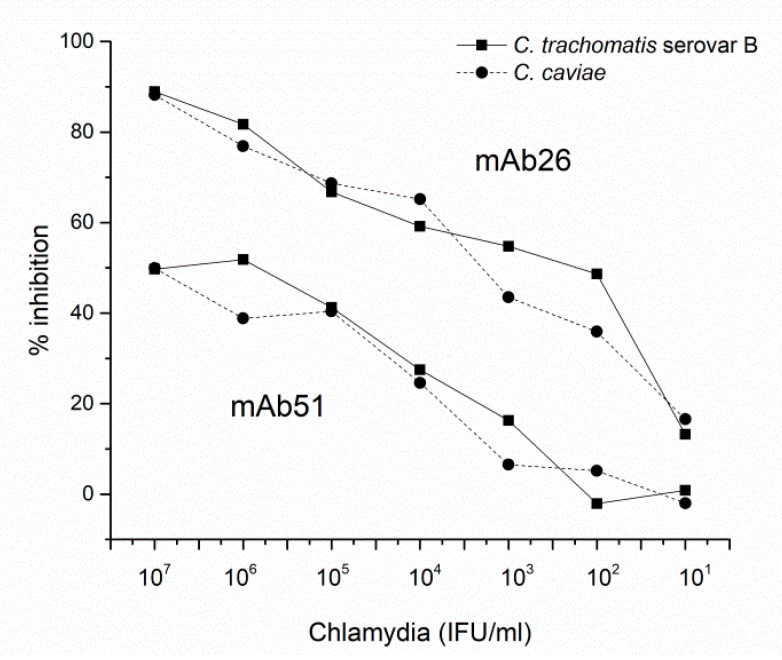
The capacity of chlamydial elementary bodies (EBs; *C. trachomatis* serovar B and *C. caviae*) to inhibit the binding of mAb26 and mAb51 to TTd. The results represent the percentage inhibition of mAb26 and mAb51 binding to TTd with chlamydial EBs: *C. trachomatis* serovar B and *C. caviae* (both: 1 × 10^7^–1 × 10^1^ IFU/mL). The percentage (%) of inhibition was calculated from recorded optical density (OD) values, taking the one recorded in wells with mAbs alone as a 100% binding.

The results of inhibitory ELISAs, where the binding of mAb26 and mAb51 to adsorbed TTd was inhibited by the preincubation with EBs of *C. trachomatis* serovar B and *C. caviae* in solution indicated differences in the binding characteristics of these mAbs to Chlamydia. Our results suggest a better inhibitory capacity of mAb26 compared to mAb51 (Figure 3).

### 3.3. The Ability of mAb26 and mAb51 to Inhibit Chlamydial Infection In Vitro

**Figure 4:**
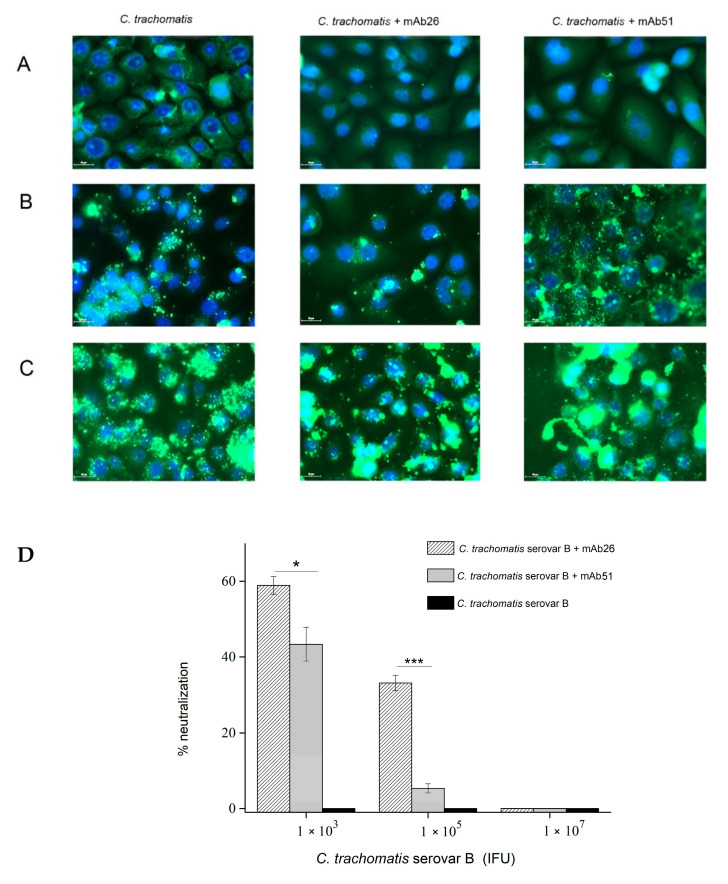
The in vitro potential of mAb26 and mAb51 to interfere with chlamydial infection in human conjunctival epithelial (HCjE) cells. Representative images of *C. trachomatis* serovar B infection of HCjE cells are presented. HCjE cells were infected with *C. trachomatis* serovar B elementary bodies (EBs) (**A**) 1 × 10^3^, (**B**) 1 × 10^5^, and (**C**) 1 × 10^7^ infectious forming units (IFU)/well previously incubated with the mAb26 and mAb51. Percentage (%) of neutralization (**D**) was determined using the equation [(number of IFU in cells incubated without mAbs-number of IFU in cells incubated with mAbs)/number of IFU in cells incubated without mAbs] × 100. The number of inclusions was visualized by fluorescent microscopy after staining with FITC-labeled anti-chlamydial LPS Abs. Samples are assessed in triplicates, and results presented as mean ± S.E: * *p* < 0.05, *** *p* < 0.001.

Our results indicate that both mAb26 and mAb51 counteract the chlamydial infection in HCjE cells, and mAb26 is far superior (Figure 4A–C). The superiority of mAb26 was particularly pronounced with increasing infectious dose. When HCjE cells were infected with 1 × 10^5^ IFU of *C. trachomatis* serovar B, the mAb26 prevented infection of 33.12% HCjE cells, while in the case of the mAb51, only 5.33% HCjE cells remained uninfected (Figure 4D). At a very high infectious dose, 1 × 10^7^ IFU, both mAbs were not capable of preventing the infection of HCjE cells. This finding, together with the results from the inhibition ELISA, showed that mAb26 can effectively neutralize chlamydial EBs in solution, leading to the inhibition of chlamydial infection in vitro.

### 3.4. The Ability of mAb26 and mAb51 to Prevent Ocular Chlamydial Infection In Vivo: A Model of Guinea Pig Inclusive Conjunctivitis

**Figure 5:**
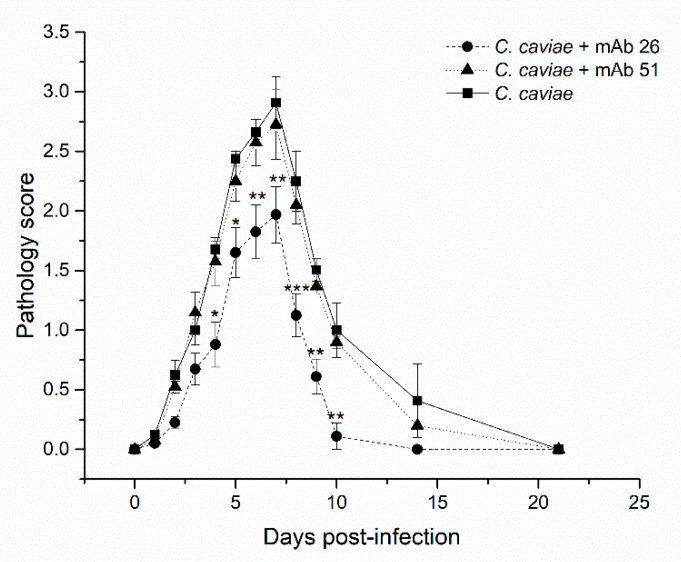
The impact of mAb26 and mAb51 on in vivo ocular chlamydial infection in guinea pigs. Guinea pigs (*n* = 5 per group) were infected (day 0) by 1 × 10^4^ infectious forming units (IFU) of *C. caviae* (preincubated without/with either mAb26 or mAb51 (10 μg/mL, 1 h/RT). Upon the infection, the local treatment with the corresponding mAb (10 μg/mL, 25 μL/eye) was performed daily until day 7 post-infection. Guinea pigs were monitored until the resolution of infection (day 21). The results represent the mean of pathology score ± S.E: * *p* < 0.05, ** *p* < 0.01, *** *p* < 0.001.

The ability of the mAb26 and mAb51 to interfere with chlamydial infection in vivo has been investigated in a model of guinea pig inclusion conjunctivitis, which is considered a relevant model for studying clinically relevant chlamydial infection. Figure 5 depicts changes in the pathology score of ocular chlamydial guinea pig infection.

**Figure 6:**
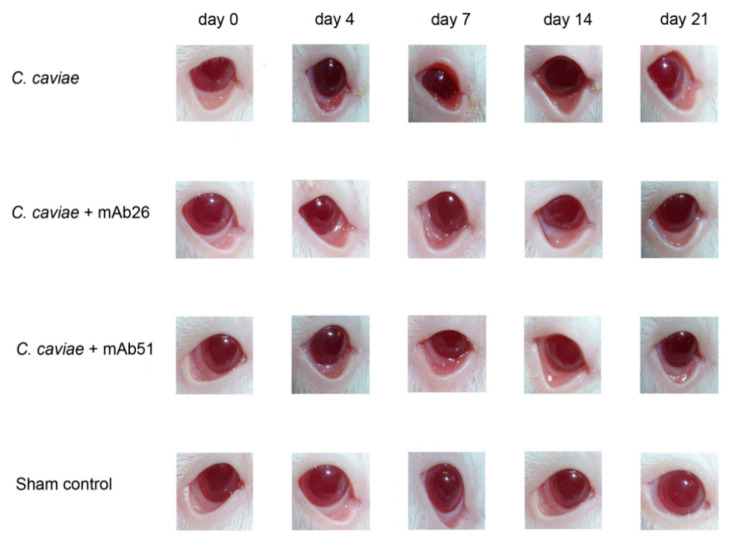
Representative photographs of guinea pigs’ conjunctiva at defined intervals (day 0, 4, 7, 14, and 21). The guinea pigs were infected with 1 × 10^4^ IFU of *C. caviae* and 1 × 10^4^ IFU of *C. caviae* preincubated with either mAb26 or mAb51 (10 μg/mL, 1 h/RT). Upon infection, the local treatment with corresponding mAb was performed daily until day 7 post-infection.

We have shown that mAb26 cannot completely prevent ocular chlamydial guinea pig infection but can significantly alleviate the intensity of pathology at the peak of the disease. Between days 2 and 9 post-infection, in guinea pigs infected with 1 × 10^4^ IFU *C. caviae*, preincubated and treated during the acute phase with 10 μg/mL of the mAb26, which is a milder clinical picture (pronouncedly less edema, redness, and conjunctival secretion) was observed compared to guinea pigs exposed to the same *C. caviae* infectious dose without the mAb preincubation or preincubated and treated with mAb51 (Figure 5). The preincubation of *C. caviae* and the follow-up treatment with mAb26 resulted in faster resolution of infection as these guinea pigs recovered by day 14 in contrast to guinea pigs infected only with *C. caviae* that recovered at the end of the follow-up period (day 21). Additionally, guinea pigs treated with the mAb26 showed a milder clinical picture of ocular chlamydial infection compared with the guinea pigs infected with *C. caviae* (Figure 6).

## 4. Discussion

Our hypothesis that heterologous immunity triggered by the tetanus vaccination could contribute to the protection against chlamydial infections is based on the literature evidence that vaccination of healthy volunteers with tetanus led to the production of chlamydia-specific antibodies when compared to non-immunized subjects [34]. Based on this assumption, we investigated whether the tetanus-specific monoclonal antibodies could bind Chlamydia, and if the binding occurs, whether they were potent enough to either neutralize and/or reduce the chlamydial infection.

The structural homology between TeNT and certain chlamydial proteins at the level of short peptide sequences implies that tetanus-specific antibodies generated upon the tetanus vaccination could potentially recognize these chlamydial proteins. In the current study, we found that the length of the peptide sequences with the best overlap was >60 amino acid residues, and the representation of identical amino acid residues was less than 30%. This finding does not diminish the possibility of recognizing chlamydial antigens by cross-reactive anti-tetanus antibodies. As it is well-known, the antibodies primarily recognize conformational, i.e., the 3D epitopes, enabling the specific establishment of hydrogen-, hydrophobic, and electrostatic interactions with a paratope of an antibody [51,52]. In other words, interactions of a cross-reactive antibody with epitopes of heterologous antigens are predominantly a consequence of electrostatic and hydrophobic homology of target epitopes, which does not require the 100% identity of amino acid sequences but the presence of amino acids with similar characteristics (positive, negative, neutral) at certain positions in the sequence [44,53,54,55]. Analysis of the contribution of individual amino acid residues to protein–protein interactions has shown that the epitope center usually consists of hydrophobic amino acids, while charged amino acid residues are located at the periphery [56]. Hydrophobic amino acid residues in the epitope center mediate and allow complex formation, while charged/hydrophilic residues keep water molecules away from the antigen–antibody contact surface and form electrostatic interactions and hydrogen bonds with the corresponding amino acid residues from the paratope allowing specific antigen–antibody recognition [55]. In addition, consideration of the phenomenon of cross-reactivity should take into account the fact that paratope hypervariance (CDR3 region) and conformational changes resulting from antigen–antibody interaction allow antibodies to recognize very different antigen surfaces and are the basis of natural strategies for the limited antibody repertoire to recognize seemingly infinite variations of protein antigens [51].

Given the role of individual proteins in the chlamydial life cycle, the existence of structural homology between TeNT and either chlamydial PmpC or MOMP-B proteins further supports the hypothesis that heterologous immunity established after the tetanus immunization could contribute to the protection against chlamydial infection. PmpC is a chlamydial adhesin that is expressed on both the RBs and EBs during the biphasic life cycle of *Chlamydiae* [43,57,58,59], and it is required for the initial interaction with the host cell [47,57,60]. Therefore, PmpC-specific antibodies can be expected to prevent/impede the initial contact of Chlamydia with the host cell and thus prevent the onset and spread of infection. This is supported by our previous research where the conjunctival immunization of guinea pigs with the N-terminal domain of PmpC expressed within probiotic bacteria *Escherichia coli* Nissle 1917 increased levels of PmpC-specific IgA in tears and reduced the intensity of ocular pathology [43]. MOMP belongs to the porin class of proteins and is necessary for the passive transport of sugars, nucleotides, as well as ions through the outer membrane of Gram-negative bacteria [61]. Further, MOMP is the most prevalent protein at the surface of Chlamydia, and, similar to PmpC, it has been shown to play a role in adhesion to the host cells [62]. Numerous studies suggest MOMP as the main antigen candidate in a future vaccine against chlamydial infections [5,18,28,29,45,63,64].

In previous studies, we have shown that the mAb26 binds tetanus with lower affinity than mAb51 and was not able to provide protection against tetanus disease [36]. On the contrary, high-affinity mAb51 was shown to be 100% protective when passively administered in mice before lethal challenge with TeNT [36]. Although mAb26 and mAb51 specifically recognize TeNT, the characteristics of their interactions with TeNT differ significantly, as do their protective potential (Table S1). After validation that the mAb26 and mAb51 could bind Chlamydia, we selected these two mAbs, possessing different binding properties to TeNT, to further explore the role of cross-reactivity in protection against chlamydial infection.

Our finding that preincubation with chlamydial EBs significantly inhibited the binding of the mAb26 to adsorbed TTd indicated that compared to mAb51, mAb26 established stronger interactions with chlamydial EBs in solution. However, a definite conclusion about the association between the chlamydia-specific neutralization potential of the mAb26 and mAb51 could not be made based on this test, given that the previously shown differences in affinities between the mAb26 and mAb51 to TeNT may contribute to this result (Table S1) [35,36].

In the context of the prevention of chlamydial infection, mAb26 was shown to be superior in both in vitro and in vivo settings compared to mAb51. In a setting that mimics the natural chlamydial infection, e.g., by using a relevant ocular epithelial cell line HCjE to investigate the ability of mAb26 and mAb51 to inhibit the infection, we showed that the mAb26 efficiently neutralized chlamydial EBs in solution and inhibited the infection in vitro. We have also shown that cross-reactive antibodies generated after tetanus immunization can interfere with chlamydial infection in vivo by alleviating the clinical picture of ocular chlamydial infection in guinea pigs. The finding that mAb26, topically applied during the acute phase of infection to the animals, was able to reduce the chlamydial infection in vivo is important and might have clinical relevance. The genital and ocular guinea pig models are established and relevant animal models to investigate chlamydial infection [43,65,66,67,68,69,70,71,72]. Furthermore, structural and functional similarity between a guinea pig and human conjunctiva makes the guinea pig infection model valuable in predicting treatment effects in humans [43,57,68,70,71].

Many processes in nature are extremely complex and very rationally designed during evolution. We suggest that certain low-affinity antibodies generated after TTd immunization, which cannot provide protection against TeNT intoxication per se, such as mAb26, could act as positive binding modulators by contributing to resistance to heterologous infectious agents. More research is needed to understand how such cross-reactive antibodies shape the chlamydial infection.

We are planning to investigate further whether the active immunization with tetanus toxoid could alleviate the chlamydial infection. Characterizing the interactions of tetanus-specific and Chlamydia cross-reactive mAbs, aiming to primarily determine their fine epitope specificity, could contribute to research aimed at formulating a vaccine that will effectively protect against chlamydial infections. Additionally, defining the precise mechanisms underlying this heterologous immunity could also pave the way to a better definition of the mechanisms that chlamydial infection exerts on specific mucosal surfaces.

## 5. Conclusions

Vaccination against tetanus disease, due to heterologous effects, might contribute to the immunity to chlamydial infections. Elucidation of mechanisms underlying heterologous impact could provide important indications for designing the efficient vaccine formulation against chlamydial infections.

## Figures and Tables

**Table 1:** Structural homology of short peptide sequences of chlamydial proteins and TeNT (UniProt ID P04958).

Chlamydial Antigens	Serovar	UniProt ID	MW (kDa)	Max Score	E Value	Nr. AA	TeNT Region with Max. Overlap
PmpC	Ct	Q6HA51	188.1	25.0	0.071	84	HC
PmpD	CtD *	O84818	160.7	20.0	1.9	40	LC
PmpH	Ct	Q2TCH7	105.6	18.1	4.3	37	HC
OmcB	CtC *	P26758	58.6	19.6	0.86	61	HC
Hsp60	CtA *	Q3KMQ9	58.1	17.3	3.6	18	LC
MOMP-A	CtA *	P23732	42.9	16.9	3.3	17	HC
MOMP-B	CtB *	P23421	42.5	24,6	0.016	60	HC
MOMP-C	CtC *	P08780	42.9	17.7	2.0	30	HC
MOMP-D	CtD *	Q46409	42.5	19.2	0.79	66	HC
MOMP-CC	CC	Q824U2	41.9	16.9	3.6	9	HC

The parameters on the BLASTp network server were set automatically. General parameters: Maximum target sequences: 100; Expected threshold: 10; Word size: 3; Maximum Matches in Query Range: 0; Scoring Parameters: Matrix–BLOSUM62, Gap Cost: Existence 11 Extension 1, Compositional Adjustments: conditional compositional score matrix adjustment. Reviewed protein sequences are indicated by an asterisk (*); Pmp (C, D, H)—Polymorphic membrane protein (C, D, H); OmcB—Outer membrane complex B; Hsp60—Heat shock protein 60; MOMP (-A, -B, -C, -D, CC)–Major outer membrane protein, Ct-*C. trachomatis*; CC-*C. caviae*; TeNT—tetanus toxin HC—heavy chain; LC—light chain.

## Supplementary Materials

The following are available online at https://www.mdpi.com/2076-393X/8/4/719/s1, Table S1: Characteristics of selected anti-tetanus mAbs.
