# Supplementary material for: Cross-Reactive Effects of Vaccines: Heterologous Immunity between Tetanus and Chlamydia
Source: Vaccines (Basel). 2020 Dec 1;8(4):719. doi: 10.3390/vaccines8040719 (PMC7712554; doi:10.3390/vaccines8040719)
Supplement: Supplementary file 1 [file vaccines-08-00719-s001.zip › vaccines-1015140-supplementary.docx]

**Supplementary Data：Table S1**

**Characteristics of selected anti-tetanus mAbs [35,36].**

| \| **mAb26** \| **mAb51** \| \| --- \| --- \| \| **1. Affinity constant and rate of establishment the anti-TeNT mAb/TeNT interaction** \| \| \| K_a_ = 0.4 ± 0.2 x 10^8^ M^-1^ \| K_a_ = 2.4 ± 0.4 x 10^8^ M^-1^ \| \| k_on_ = 1.1 ± 0.2 x 10^3^ M^-1^ s^-1^ \| k_on_ = 185 ± 16 x 10^3^ M^-1^ s^-1^ \| \| Low affinity mAb \| High affinity mAb \| \|  \|  \| \| **2. Epitope positions on TeNT** \|  \| \| Epitopes for mAb26 and mAb51 are located on close proximity of the ganglioside-binding place \| \| \|  \|  \| \| **3. Capability to inhibit TeNT/GD1b interactions** \| \| \| Limited inhibition of TeNT/GD1b interactions \| Complete inhibition of TtNT/GD1b interactions \| \| (29.9 ± 3.1 %) \| (98.8 ± 1.1 %) \| \|  \|  \| \| **4. *In vivo* protective potential against TeNT intoxination** \| \| \| Not protective against TeNT intoxination \| Fully protective against TeNT intoxination \| |
| --- | --- | --- | --- | --- | --- | --- | --- | --- | --- | --- | --- | --- | --- | --- | --- | --- | --- | --- | --- | --- | --- | --- | --- | --- | --- | --- | --- | --- | --- | --- |
